# PhenomeExpress: A refined network analysis of expression datasets by inclusion of known disease phenotypes

**DOI:** 10.1038/srep08117

**Published:** 2015-01-29

**Authors:** Jamie Soul, Timothy E. Hardingham, Raymond P. Boot-Handford, Jean-Marc Schwartz

**Affiliations:** 1 Wellcome Trust Centre for Cell-Matrix Research, Faculty of Life Sciences, University of Manchester, Manchester M13 9PT, UK

## Abstract

We describe a new method, PhenomeExpress, for the analysis of transcriptomic datasets to identify pathogenic disease mechanisms. Our analysis method includes input from both protein-protein interaction and phenotype similarity networks. This introduces valuable information from disease relevant phenotypes, which aids the identification of sub-networks that are significantly enriched in differentially expressed genes and are related to the disease relevant phenotypes. This contrasts with many active sub-network detection methods, which rely solely on protein-protein interaction networks derived from compounded data of many unrelated biological conditions and which are therefore not specific to the context of the experiment. PhenomeExpress thus exploits readily available animal model and human disease phenotype information. It combines this prior evidence of disease phenotypes with the experimentally derived disease data sets to provide a more targeted analysis. Two case studies, in subchondral bone in osteoarthritis and in Pax5 in acute lymphoblastic leukaemia, demonstrate that PhenomeExpress identifies core disease pathways in both mouse and human disease expression datasets derived from different technologies. We also validate the approach by comparison to state-of-the-art active sub-network detection methods, which reveals how it may enhance the detection of molecular phenotypes and provide a more detailed context to those previously identified as possible candidates.

Transcriptomics technologies such as RNA-Seq and microarray are invaluable in the study of human disease by identifying differentially expressed genes between experimental groups^1^. Interpreting these large datasets is time consuming, since many significantly differentially expressed genes may be detected. It is difficult to identify the genes and biological pathways most relevant to the disease in an unbiased fashion to formulate a biological hypothesis for further experiments. The simplest approach of ranking the genes by fold change between experimental conditions or by statistical significance may miss lower ranked, but functionally critical genes.

Multiple gene prioritisation tools for expression data have been developed that focus on ranking genes with the aid of integrated information such as protein interaction or gene ontology data^2^. A common approach is to overlay the expression information onto a protein-protein interaction (PPI) network. One tool, PINTA, uses a random walk with restart based algorithm to rank differentially genes on a protein interaction network by their ‘influence impact’ in the network^3^.

Another often used approach is to identify de novo sub-networks or pre-defined pathways that are enriched in differentially expressed genes. Interacting differentially expressed genes can give stronger evidence of an altered biological process, even if the individual genes are not statistically significant in isolation^4^. The tool JActivemodules uses a simulated annealing approach to find these hotspots of differentially expressed genes, while GIGA uses iterative addition of high scoring nodes to form sub-graphs^5^,^6^. BioNet transforms the problem to that of the prize-collecting Steiner tree problem to find the best path to high scoring nodes and provides a heuristic to identify the maximum scoring sub-network^7^.

These tools use a protein interaction network derived from many cell types and biological conditions. The resulting generic PPI network therefore lacks biological context and contains interactions not relevant to the biological condition under study. Integration of additional biological information to these generic PPI networks is a powerful approach to regain lost biological context. For instance, construction of tissue specific PPI network by removal of proteins whose encoding genes are not expressed in the tissue under study, has been shown to improve network-based gene prioritisation algorithms^8^. Sub-network detection methods which utilise just the topology of the PPI network and the overlaid expression data may not identify the regions of the interactome most relevant to a disease. Further integration of disease related information could guide identification of the most biologically important regions of the interactome with regards to a disease transcriptomics dataset.

One resource that has not been well utilised in transcriptomics analysis is model animal phenotype information. Higher order animal models such as zebrafish and mouse are invaluable for studying protein function in a complex in vivo environment, relevant to human disease. Many genes have been knocked-out in a low throughput manner and the resulting phenotypes reported in the literature. More recently, consortiums have begun systematically knocking out all genes in mouse and other model organisms for phenotype screening in a high throughput manner. This will eventually lead to a complete resource of gene to phenotype associations^9^,^10^. The observed phenotypes are recorded in phenotype ontologies with a controlled vocabulary that allow standardized reporting^11^,^12^.

Orthologous genes that have a similar phenotype from mouse to human have been invaluable for the study of rare monogenic human disease^13^. The use of cross-species phenotypes increases the coverage of gene to phenotype associations. It has been demonstrated that the associated mouse phenotypes of a human gene ortholog are a better predictor of perturbed human gene phenotypes than gene ontology or pathway based measures^14^. In addition, if multiple species have a similar phenotype upon gene perturbation this presents greater evidence of a fundamental, conserved mechanism that may be of relevance to disease. Therefore, it is valuable to translate cross-species phenotypes to human by finding equivalent phenotypes between species specific ontologies. A cross-species ontology has been constructed from species-specific ontologies though an automated reasoning approach, allowing the mapping of phenotypes across species, thus facilitating the inference of disease related genes^15^. Approaches are being developed to exploit these phenotype-gene association resources to apply to human disease diagnostics and gene variant prioritisation^16^.

Several informatics tools have previously used disease phenotype information from Mendelian human disorders to add additional power to generic gene to disease association and prioritisation. Li *et al*. used a random walk based algorithm on a heterogeneous network comprised of a human disease phenotype similarity network connected to a protein interaction network by known disease to gene associations^17^. This was used to prioritise a set of disease genes related to a given phenotype. Similarly, another method MAXIF uses a maximal flow propagation method to rank known gene disease associations^18^. It was noted that highly ranked genes for a disease tend to be in close proximity in the interactome, thus supporting the idea of sub-networks of important disease genes. An important feature of these algorithms is the inference of related phenotypes to the selected seed phenotypes. In these studies, the phenotype interaction network was derived from text mining of OMIM clinical synopsis free text which uses an uncontrolled vocabulary, resulting in potentially spurious interactions between phenotype terms. A more recent approach, PhenomeNET used a cross-species phenome network to rank candidate human disease genes^19^. These approaches, whilst useful, do not show the predicted driver genes in the context of the interactome and how they interact. Furthermore, these approaches have not been applied to disease related transcriptomics data to identify and visualise biologically important sub-networks of differentially expressed genes.

In this work we aim to effectively combine these ideas to create a tool that allows detection of sub-networks relevant to the disease of interest. Our method, PhenomeExpress, utilises both the transciptomics data being analysed and the prior knowledge of all cross-species phenotype to gene associations, including those directly related to the disease understudy. We adapt and combine existing algorithms for node ranking and sub-network detection with a cross-species phenome-interactome network to automatically detect sub-networks of proteins from transcriptomics data that are relevant to the disease of interest. We present two case studies with human microarray and mouse RNA-seq data to show the utility of our tool and compare the results to that of state-of-the-art sub-network detection algorithms.

## Results

### Overview of the algorithm

**Figure 1:**
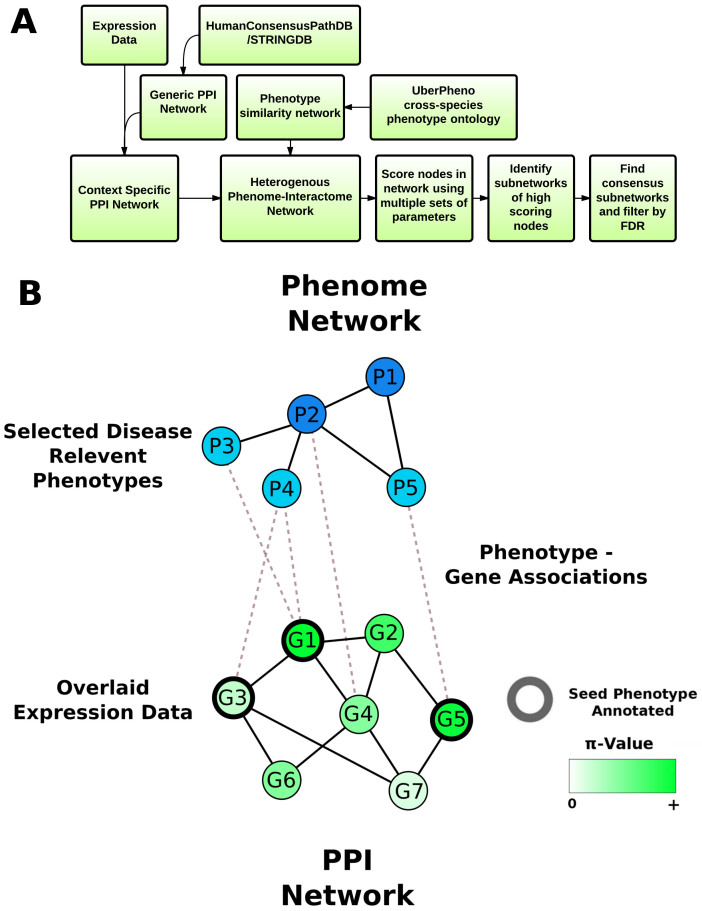
Summary of Phenome Express. (A) Expression data is analysed to compute the π-value and to determine which genes are expressed. This is overlaid onto a PPI network and connected to a phenotype-phenotype similarity network via phenotype to gene associations. The nodes in the PPI network are scored by a random walk based method with multiple sets of parameters. High scoring sub-networks are then extracted from these scored networks. Consensus sub-networks are generated and filtering is performed by FDR analysis with random sub-network sampling. (B) The heterogeneous network consists of a protein-protein interaction network (PPI) and a phenotype-phenotype similarity network connected by known phenotype to gene associations. Seed phenotypes (light blue) relevant to the disease under study are selected. Genes directely associated with these seeds phenotypes are indicated with a black node border. The π-values from the expression data is overlain onto the PPI network (green gradient). The topology of the phenome network and PPI allows inference and scoring of disease relevant genes from the transcriptomics study with the random walk based algorithm.

Our algorithm aims to find sub-networks of proteins that are enriched in differentially expressed genes and genes that are directly/indirectly related to the known phenotypes of the disease, so as to produce disease relevant sub-networks specific to the transcriptomics experiment being analysed (Figure 1A). We use a two stage algorithm where proteins in a PPI network are first scored on the basis of their topology in the network with regards to the differential expression and known/inferred phenotype annotated. In the second stage of the algorithm, sub-networks of highly scoring nodes are identified and assessed for statistical significance.

### Construction of the cross-species phenome to interactome heterogeneous network

Prior to running the algorithm, a heterogeneous network is constructed, comprised of a phenome (phenotype to phenotype) network and a PPI network connected by known phenotype to gene associations (Figure 1B). We use experimentally derived PPI networks and remove all proteins not expressed above the experimental background in the transcriptomics experiment in order to create a context specific PPI network. This approach has previously been shown to improve performance in gene prioritisation approaches with random walk based algorithms^8^. The phenome network allows the integration of phenotype information with the PPI network. The phenome network was constructed from the UberPheno cross-species ontology which includes human, mouse and zebrafish phenotypes. Resnik semantic similarity between the ontology terms was used as a measure of similarity between phenotypes and was used to weight the edges in the resulting phenome network. A semantic similarity threshold of 3 was used to keep only those interactions that are relatively specific and more likely to be biologically meaningful. The human disease and animal model derived phenotype to human gene associations were retrieved from UberPheno and used to connect the phenome and protein interaction networks.

As a preprocessing step, the transcriptomics data is analysed to produce the fold change for each expressed gene and the statistical significance. An established approach of combining the fold change and the statistical significance into one continuous score (π-Value) which reflects both the biological significance (fold change) and the statistical significance (multiple testing adjusted p-value) was used^20^. Continuous scores have previously been shown to provide more accurate results with random walk with restart based algorithms^21^. This approach also avoids the need for an arbitrary cut-off of fold change/statistical significance to define differentially expressed genes.

### Algorithm description

A random walk on a heterogeneous network (RWHN) algorithm has been previously described in detail for use of identifying novel disease genes by inference from known disease associated genes. We use this approach to score proteins in the context specific protein interactome using the entire topology of the heterogeneous network. In the algorithm, a random walker moves from node to node in the PPI or phenome networks with a probability equal to the edge weight (interaction confidence or semantic similarity score). With each movement between nodes, there is a probability (α) of restart/teleporation to another node in the network. Additionally, at a node where there is an edge between the phenome and PPI network there is a probability (λ) of transitioning from the phenome to PPI and 1- λ from the PPI to the phenome.

The probabilities of restarting on each node in the heterogeneous network are given by a vector of probabilities. In the phenome network, the probability of restarting is equal among the predefined ‘seed’ phenotypes, which reflect the known phenotypic characteristics of the disease under study. For the PPI network, the restart can occur on any protein in the network, with a probability that is proportional to the π-value of the encoding gene from the transcriptomics data. The relative weighting of the total phenotype and protein restart probabilities in the teleportation vector is given by the parameter η. We perform a random walk with restart on the heterogeneous network over a range of parameters so as to maximise the chance of finding relevant significant sub-networks downstream of the scoring stage. The nodes are automatically scored with all combinations of parameters within the range α 0.2–0.8, η 0.2–0.8 and λ 0.5–0.8 with 0.1 steps. The parameters were chosen to avoid extremes while covering a wide range, and were found empirically to produce biologically informative sub-networks with a range of transcriptomics disease datasets. The transition probability, λ, was set to be at least 0.5 to promote connectivity between the two networks. The resulting scores of the proteins are used to produce a ranked list of proteins for each combination of parameters. All 196 ranked lists from the different parameter combinations are used in the second stage of the algorithm.

Highly ranked proteins will tend to be differentially expressed or related to the seed disease phenotypes. In the second stage of the algorithm we therefore identify sub-networks of these highly ranked proteins. For sub-network detection we employ the previously described GIGA algorithm^6^. Briefly, GIGA takes a PPI network and a ranked list of nodes as input. GIGA then identifies the locally lowest ranked proteins in the PPI network and iteratively adds higher ranked nodes that are directly connected and any surrounding nodes of lower rank to the resulting sub-network. At each step the statistical significance of the new sub-network is assessed. The iterations continue until the addition of a higher ranked node and any surrounding nodes of lower rank no longer improves the significance of the sub-network, or the sub-network reaches a pre-defined maximum size. The most significant sub-networks are subsequently outputted.

GIGA is run on each ranked list from the first stage of the algorithm to identify high-scoring sub-networks of a user defined maximum. Larger consensus sub-networks are generated by including only those proteins that have complete co-occurrence in all sub-networks across the range of parameters. As a final step, empirical p-values are calculated by sampling random sub-networks. This allows filtering of those sub-networks significantly enriched in differentially expressed genes. For each sub-network we sample 10000 random networks of equal size and compare the sum of the π-values in the sub-networks with those observed. The empirical p value is calculated by: 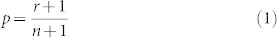
where *r* is the number of random sub-graphs with a total π-value greater or equal to that of the extracted sub-graph and n is the total number of random sub-graphs. This stage ensures that the resulting consensus sub-networks are unlikely to be detected by chance and also removes those sub-networks that are not strongly related to the transcriptomics study.

### Experimental Case Study 1 – Subchondral bone in osteoarthritis

To demonstrate the utility of our method in finding disease relevant sub-networks from transcriptomics data an osteoarthritis related dataset was analysed. Osteoarthritis (OA) is a joint disease characterized by degradation of the collagen/proteoglycan extracellular matrix that comprises the articular cartilage, and structural changes in the subchondral bone^22^. There is increasing evidence that OA is a whole joint disease, with gene expression changes observed in all components of the joints of OA patients, even in visibly undamaged regions^23^,^24^. A change in phenotype of the chondrocytes that regulate extracellular matrix metabolism in the articular cartilage is thought to result in the observed cartilage degradation^25^. Similarly, osteoblasts and osteoclasts that act to control bone ossification and resorption in the subchondral bone, respectively, have been shown to have altered phenotypes in OA, and these changes are also thought to contribute to the disease pathogenesis^26^.

An existing microarray dataset comparing macroscopically normal knee lateral tibial bone from healthy and OA patients was analysed^23^. The fold changes and statistical significance in genes between the two groups were used to calculate the π-value for each gene. Expressed genes in the dataset were identified by finding those genes with corresponding probe intensity above the chip background probes. The baseline protein interaction network derived from HumanConsensusPathDB was then filtered to remove non-expressed proteins and create a tissue specific protein interaction network^27^.

The Phenomiser tool, which list phenotype annotations of OMIM diseases, was used to aid the selection of the seed phenotypes that are related to the observed bone phenotypes in OA and OA disease terms^28^. From this and through manual searching of the ontology for relevant terms, 8 bone/OA related phenotypes were selected, which had a total of 238 associated proteins that were present in the filtered network (Table 1). The tool was applied to score the proteins in the network using a range of parameters, and identify initial sub-networks of maximum 15 proteins. These small sub-networks were merged to form consensus sub-networks and subsequently filtered by a FDR of 5% with sampling of 10000 random sub-networks to calculate empirical p-values, resulting in 11 sub-networks summarised in Table 2.

**Figure 2:**
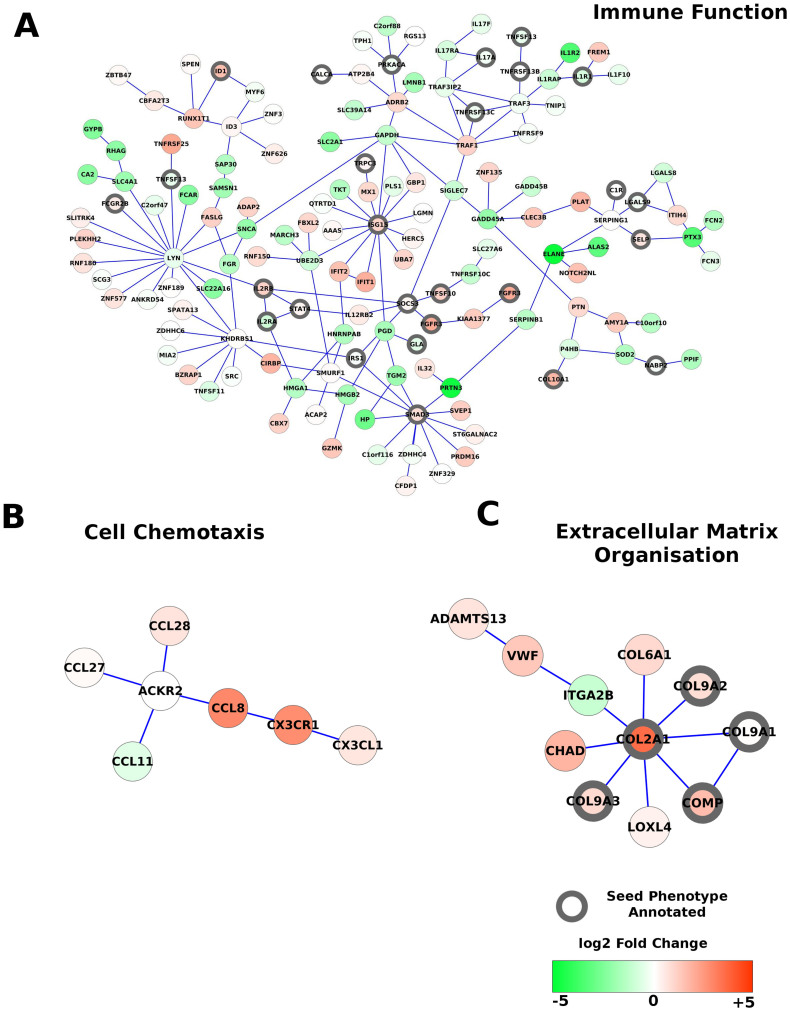
Bone sub-networks in osteoarthritis. Visualization of selected sub-networks identified by PhenomeExpress with the subchondral bone dataset. Proteins annotated with one of the seed phenotypes are indicated with a black node border. Nodes are coloured by their fold change relative to healthy bone.

The largest sub-network identified by our tool contains 145 proteins of which 27 are directly associated with a seed phenotype (Figure 2A). This sub-network is highly enriched in differentially expressed genes with no random sub-network having a total π-value equal or greater that our sub-network (Supplementary Figure S1). Gene ontology annotation of this sub-network showed that it is enriched in proteins related to immune function including TNFRSF10C, ELANE and IL2RB. Several differentially expressed genes are annotated to a seed phenotype. For instance, the proteins TNFS10, SMAD3, ISG15 are annotated to increased susceptibility to induced arthritis, knee OA and abnormal bone ossification phenotypes respectively, suggesting the phenotype information complements the expression information. Interestingly, altered pro- and anti-inflammatory processes have been reported in the subchondral bone of a surgical mouse model of early OA^29^. This sub-network supports the idea of an altered inflammatory process in OA subchondral bone.

In this sub-network, several of the proteins annotated with OA associated phenotypes, which are therefore of potential interest, are not the most differentially expressed, implying they may be excluded by simple fold change cut-off approaches. For instance, SOCS3, which is not strongly differentially expressed (1.3 fold) is known to regulate both pro-inflammatory signals and bone remodelling by osteoblasts/osteoclasts and interacts with several more strongly differentially expressed genes^30^,^31^. Therefore, by including the phenotype associated gene information additional relevant genes will be considered for inclusion in the sub-networks.

Related to the inflammation sub-network, another significant sub-network contained several cytokines and cytokine receptor proteins (Figure 2B). Cytokines and their receptors act to recruit immune cells to inflamed tissue^32^. These proteins are implicated in OA and have been suggested to be potential therapeutic targets. CXCL1 expression by osteoclasts has been shown to induce osteoclast precursor migration and differentiation via binding to its receptor CX3CR1, resulting in bone remodelling^33^. In a transcriptomics study of site-matched articular cartilage and underlying subchondral bone, expression of CCL8 was found to be significantly correlated with severity of OA cartilage degradation and subchondral bone remodelling, suggesting it may be important in the pathogenesis of the disease^34^. The sub-network contains no seed phenotype annotated proteins, suggesting the method can find relevant sub-networks by inference and the expression data alone. Several other sub-networks annotated to inflammatory processes such as positive regulation of mast cell cytokine production, and defense response to fungi were also identified.

Another sub-network identified by our method contains extracellular matrix proteins including genes encoding Collagen II, Collagen VI, Collagen IX (Figure 2C). Collagen II is a major component of the extracellular matrix in articular cartilage which is degraded in OA. Its expression is generally associated with chondrocytes, but it has been suggested that mesenchymal cells in bone express Collagen II which is then transported to the cartilage^35^. In the subchondral bone of a surgical mouse model of early OA increased COL2A1 expression was also observed^29^. The authors suggest a model where Collagen II induces cytokine production and metalloprotease production in the overlying cartilage. The cytokine signalling and collagen related sub-networks described here are consistent with the idea of the altered extracellular matrix and inflammatory processes in the subchondral bone contributing to the pathogenesis of the disease.

**Figure 3:**
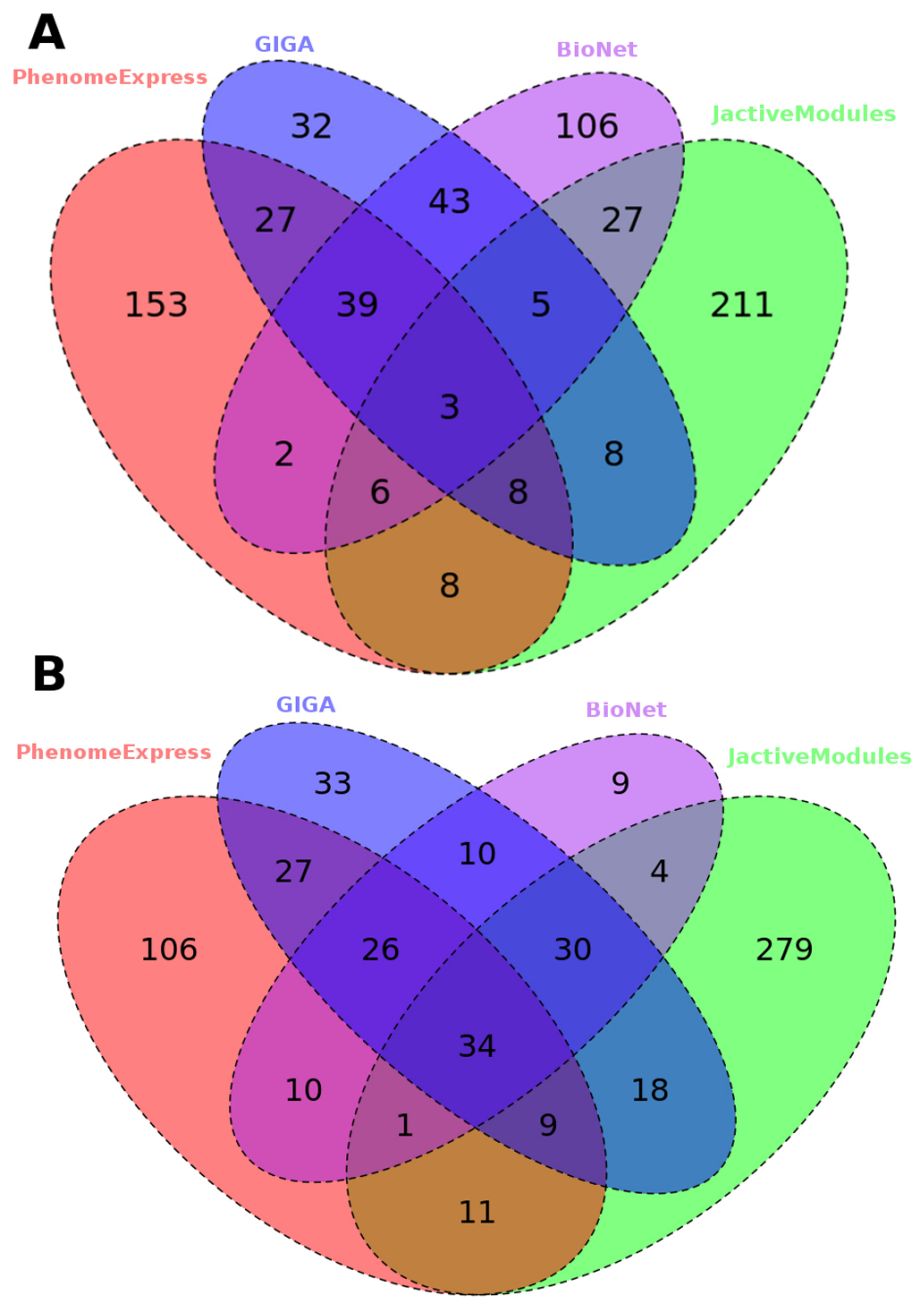
Comparison of active sub-networks identified by different algorithms. The OA (A) and PAX5 (B) datasets was analysed with PhenomeExpress, BioNet, GIGA, JActivemodules. The overlap of proteins present in sub-networks between the tools is shown as a Venn diagram.

To compare our results against methods that do not use phenotype information, JActivemodules in simulated annealing mode, GIGA and BioNet were used to generate sub-networks from the same processed dataset and protein interaction network. BioNet finds the largest maximum scoring sub-network given a FDR threshold. The FDR was chosen to give the sub-network that was most similar in size to the largest sub-network identified by our method. The resulting sub-networks are summarised in Table 2. JActivemodules identified 7 sub-networks of at least 5 proteins, from three separate runs with random seeds, while GIGA identified 5. Venn diagrams of all proteins present in significant sub-networks revealed strong differences in the results from the tools (Figure 3A). The majority of proteins present in the sub-networks from BioNet, JActivemodules and our method were not identified by another tool. GIGA mainly found proteins covered by the other tools. For instance, GIGA identified a small sub-network of extracellular matrix proteins (network 3) which overlapped with our more complete ECM sub-network. Despite the differences in the proteins identified, GIGA and JActivemodules also identified sub-networks annotated to immune process such as neutrophil aggregation and regulation of cytokine mediated signalling pathway.

The inclusion of seed phenotype annotated proteins differed between the tools with 3% (7/231) in BioNet, 3% (9/285) in JActivemodules and 4% (7/165) in GIGA compared with 21% (52/246) in our method. These results suggest, that as anticipated, our method generates sub-networks enriched with the seed phenotype annotated proteins compared to methods that do not use that information. These directly annotated proteins are likely to be of interest for identifying a potential disease mechanism from the expression data. Our method therefore provides another important angle for interpretation of the data.

### Experimental case study 2 – The role of PAX5 in pre-B cell acute lymphoblastic leukaemia

Mice are often used in disease mechanism studies as a model organism. To demonstrate the use of our tool on mouse data we took a recent RNA-Seq dataset examining the role of the transcription factor PAX5 in pre-B cell acute lymphoblastic leukemia (B-ALL)^36^. PAX5 is a master regulator of B-cell lineage programs from progenitor cells and regulates expression of B cell specific genes^37^. PAX5 is often mutated in B-ALL, resulting in loss of function and therefore a blockage of differentiation during B-cell development^38^. A mouse model with inducible suppression and restoration of PAX5 expression in B-cells was used to examine if rescue of endogenous PAX5, after loss of expression, is sufficient to return mice to a normal phenotype. The authors observed a rescue of B-ALL mice upon expression of PAX5, with recovery of the differentiation process, normal B cell receptor development and cell cycle/DNA replication suppression. Transcriptomics analysis of this model was used to understand the transcription response to PAX5 restoration in B-ALL leukaemia cells.

Differentially expressed genes were identified and the expressed genes (FPKM > 2) were used to produce to a cell specific protein interaction network. The phenotype- human gene annotations were mapped to mouse orthologs to allow integration with the STRINGDB derived mouse protein interaction network^39^. Five specific phenotypes which describe the key features of B-ALL were selected as seed phenotypes, which are annotated to 188 genes present in our network (Table 3).

**Figure 4:**
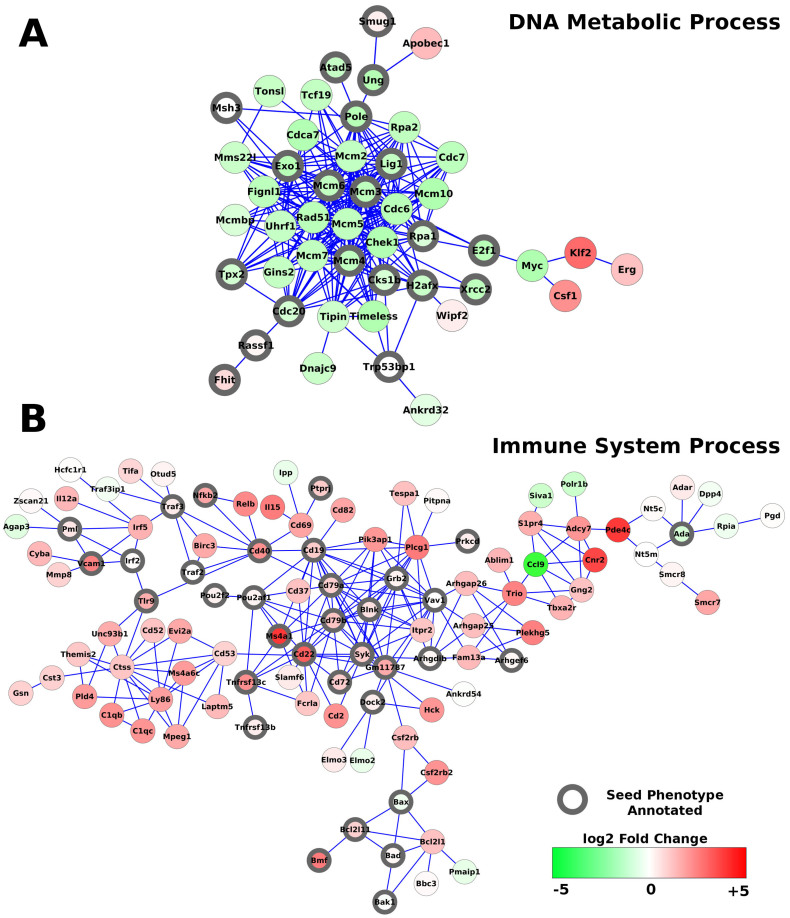
PAX5 related sub-networks in B-ALL. Visualization of selected sub-networks identified by PhenomeExpress with the PAX5 B-ALL dataset. Proteins annotated with one of the seed phenotypes are indicated with a black node border. Nodes are coloured by their fold change relative to before PAX5 expression.

Using a maximum initial sub-network size of 20 proteins, our tool identified 8 significant sub-networks summarised in Table 4. We also analysed the dataset with the other sub-network methods as before (Table 4). The results were more similar between the tools than the OA dataset, suggesting a stronger core biological signal (Figure 3B). Sub-network 2 from our method has GO annotation of DNA metabolic processes (Figure 4A). Sub-networks related to the cell cycle were also identified by the other tools (Table 4). The strong down regulation of proliferative genes was reported to be a major feature of the rescue with PAX5 expression^36^. This suggests that multiple methods with distinct approaches can find sub-networks with similar biological functions. As before our sub-networks contained many more seed phenotype annotated genes than those method that do not use that information with 25% (55/224) in PhenomeExpress, 10% (39/386) in JActivemodules, 16% (20/124) in BioNet and 12% (24/195) in GIGA.

GO analysis of the largest sub-network (sub-network 1) revealed it contains proteins annotated to immune response and the pre-BCR receptor including CD22 which is a co-receptor that mediates signalling cascades upon BCR receptor ligation (Figure 4B)^40^. This sub-network is consistent with the reported increased BCR receptor assembly follow PAX5 expression. Those genes in this sub-network annotated with seed phenotypes that are differentially expressed include BLNK, which is highlighted in the dataset's corresponding publication, but not included in sub-networks from the other methods. BLNK is a central coordinator of signalling pathways downstream of the BCR receptor and important in B cell development^41^. B-ALL spontaneously develops in BLNK-deficient mice as it has a critical function in the pro-B cell to pre-B cell transition^42^. These results suggest that including disease phenotype information focuses the sub-network on regions of interest to the disease.

The BioNet sub-network contains only differentially expressed genes with the exception of two; Crebbp and Notch1. Clearly, not all components of biological pathway have to be differentially expressed for that region of the interactome to be of interest. For instance, in the largest sub-network is a group of proteins which includes the non-differentially expressed BAD, BAX and BAK and the differentially expressed BCL2L11, BCL2L1 and BMF. GO enrichment analysis of this group showed the proteins are related to cellular death. Apoptosis is a major factor in the maintenance of normal cell count and modulators of this pathway are of therapeutic interest in cancers^43^. PAX5 expression in vitro is known to induce cell death and is a known regulator of p53, which influences cell death pathways^44^,^45^. Several of the proteins in thus group are directly annotated to the seed phenotypes and BCL2L1 is annotated to T cell related phenotypes such as impaired T cell function which are semantically similar to the seed phenotypes. This group of proteins was not identified by the other methods, suggesting our method can provide additional biological context for transcriptomics analysis. Analysis of this dataset with our sub-network tool shows the key expression changes in the driving pathways of B-ALL leukaemia and summarises the altered cellular processes that were reported rescued by the expression of PAX5.

## Discussion

Transcriptomics is often used to identify differentially expressed genes to help elucidate the mechanism of action in a disease and to ultimately aid development of rational, targeted treatments. Existing active sub-network detection tools do not take advantage of the wealth of phenotype information available to improve the understanding of disease etiology. Mouse and zebrafish gene phenotype resources are continuously growing, so there is a clear need for tools that utilize this resource for transcriptomics analysis. To our knowledge this is the first tool for disease sub-network detection on the basis of transcriptomics and cross-species phenotype data. PhenomeExpress automatically extracts the relevant regions of the protein interactome, utilising the transcriptomics data and the prior knowledge of existing disease related phenotype associated genes, as well as those inferred from the topology of the heterogeneous network.

Our method has an important advantage over existing methods in allowing injection of biological context into the sub-network identification process, by integration of prior knowledge. Therefore, it increases the likelihood of detection of biologically relevant sub-networks of interest to the researcher. This is an improvement on “unbiased” approaches, where the inclusion of various annotated evidence, based on unfiltered sources of mainly irrelevant origin, would seem more likely to cloud the analysis and make a high threshold for the discovery of relevant nodes/pathways. It is important to note that the genes annotated to the observed seed phenotypes are not necessarily annotated as been involved in the disease under study, for example, if the original study reporting the phenotype examined another disease. In addition, even though the disease displays a phenotype, not every gene annotated to the phenotype will be directly involved in the disease, as there are possibly multiple pathways controlling the same phenotype, which can be perturbed in disease. The phenotype-gene association information helps guide the algorithm to the regions of the interactome that are likely to be perturbed and the expression data helps score the most relevant genes.

In the distinct case studies presented, PhenomeExpress clearly highlights sub-networks of interest that are suitable for critical evaluation and experimental exploration. As opposed to a method which just uses the direct phenotype related proteins as seeds to be explicitly included in sub-networks, our method avoids this bias and allows the topology of the phenome to identify related phenotypes from the phenotype seeds and thus infer the most relevant proteins for inclusion in the sub-networks. Results from existing sub-network methods can be integrated with phenotype information by post-identification overlay, but this does not take advantage of the associations of related phenotypes in the initial identification of the sub-networks. In our method, genes that are only moderately differentially expressed, but directly/indirectly associated to the seed phenotypes can potentially be high scoring, thus likely to be included in a sub-network. It is important to note that PhenomeExpress did not cover all disease relevant sub-networks found by the other tools. For example, in the cation transport related network 7 from GIGA, in the B-ALL dataset, several V-type ATPases are included which are potential drug targets to increase sensitisation of cancer cells to chemotherapeutics^46^. We therefore recommend the use of PhenomeExpress as an additional tool to gain a different perspective for analysis using the awareness of the phenotypic information. One drawback of using a PPI network approach is the reliance on accurate interaction information. The PPI network could be replaced with a co-expression networks derived from the transcriptomics data which may allow for more complete coverage of the proteome.

Our method can ease translation of transcriptomics data into biological hypotheses which is a key, challenging step. We show the method generates biologically significant sub-networks which are enriched in disease phenotype associated genes. In our two case study datasets we demonstrate that PhenomeExpress can extract sub-networks relevant to the core disease processes, that include proteins associated with the known disease phenotypes and are supported by the literature. This tool can be used in conjunction with examining the most highly ranked genes by fold change, other sub-network detection methods, as well as using predefined pathways and GO enrichment analysis.

## Methods

### Phenome network construction

The UberPheno ontology and annotated human genes were downloaded from 29/04/14^47^. The Java OWL API was used to parse the ontology and calculate the lowest common submer between ontology terms^48^. Subsequently, the information content was calculated from the gene to phenotype annotations and the semantic similarity calculated as the highest information content of the lowest common submer.

### Transcriptomics Dataset Preprocessing

The gene expression case study datasets GSE51588 and GSE52870 were downloaded from the Gene Expression Omnibus (GEO)^49^. The microarray data was normalised and analysed with the Bioconductor packages lumi and limma^50^,^51^. The RNA-Seq counts previously mapped and quantified by the authors with subread and featureCounts respectively were used to detect differentially expressed genes with DESeq2^52^,^53^,^54^. For the microarray data genes that had an intensity of >5 in more than half the samples were regarded as been expressed. For the RNA-seq dataset a cutoff of FPKM (using effective gene length) of >2 was used (chosen from the binomial distribution of the FPKM values).

### Random walk on a heterogeneous network

The transition matrix for scoring the heterogeneous network in the first stage of the algorithm was constructed similarly to previously described^17^. The transition matrix of the heterogeneous network (M) is comprised of the sub-graph transition matrix of the PPI network (*M_G_*) and the Phenome network (*M_P_*), as well as the inter-sub-graph transition matrices (*M_GP_* and *M_PG_*): 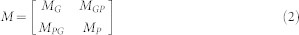
The inter-sub-graph matrices of transition probability from the i-th gene to the j-th phenotype, and vice-versa, are calculated as: 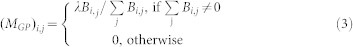

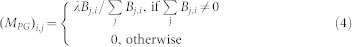
where *B* is the bipartite graph of gene to phenotype associations and *λ* is the probability of movement between the PPI and phenome networks.

*M_G_* gives the probability of transition between the genes at the *i*-th row and the *j*-th column and is the calculated from the confidence weighted adjacency matrix of the protein-protein interactions (*A_G_*): 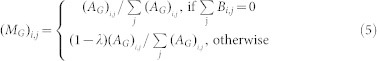
Similarly, the phenome transition matrix *M_P_* gives the probability of transitioning between phenotypes at the *i*-th row to the *j*-th column of the sematic similarity weighted phenotype adjacency matrix (*A_P_*): 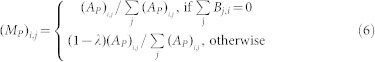
where *A_P_* is the phenome adjacency matrix weighted by the semantic similarity between the phenotype terms.

The initial probability vector for the PPI network gives the starting probability of finding the random walker at each node. This is set to be proportional the π-value for each gene such that the total probabilities sum to *η*. The initial probability vector for the phenome network is given equal among the seed phenotype nodes such that the total probability sums to *1-η*. The two probability vectors are combined to give the initial probability vector *p_0_*. The probability of finding the random walker at each node in the steady state is calculated using the iterative equation: 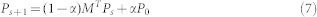
where *p_s_* is the probability of finding the random walker at node *i* at step *s*, *M_T_* is the transpose of transition matrix *M* and *α* is the probability of restart. The iterations continue until the difference between *P_s_* and *P_s+1_* is less than 10E-6.

### Protein interaction data

Protein interaction networks were derived from HumanConsensusPathDB (v28) and StringDB (v9.1) for human and mouse respectively^27^,^39^. The STRINGDB dataset was filtered to exclude low confidence (score <0.7) interactions, since it includes potentially spurious text mining derived results. To create tissue specific versions of the protein interaction networks the proteins that were not identified as being expressed were removed. The giant connected component was used as the tissue specific network.

### Validation of sub-networks

Bingo was used to assess significantly enriched pathways using the Uniprot Gene Ontology annotations for mouse and human (release 08/07/14)^55^. Networks were visualised in Cytoscape^56^. JActivemodules (v1.8) in simulated annealing mode was used with the expression datasets p-values and default settings in three runs with random seeds^5^. On each run the largest network was analysed recursively until it reach a size comparable to the largest sub-network detected by our method. BioNet (v1.23.2) was used with the fastHeinz approximation method with a FDR of 1E-5 and 1E-25 for the human and mouse datasets to generate similar sized modules to the other tools for more direct comparison^7^. Similarly, GIGA was used with the calculated π-values and with the maximum size of the sub-networks set to the largest sub-network identified by our tool^6^.

### Availability

The R source code of PhenomeExpress and scripts used for the analysis of the case studies are available at https://github.com/soulj/PhenomeExpress

## Supplementary Material

Supplementary Information Supplementary Figure S1

## Figures and Tables

**Table 1:** Phenotypes selected for osteoarthritic subchondral bone. UberPheno phenotype terms selected for use in the sub-network detection with the OA subchondral bone dataset

Phenotype ID	Phenotype Name
HP:0005086	Knee osteoarthritis
MP:0003724	Increased susceptibility to induced arthritis
HP:0002829	Arthralgia
HP:0100777	Exostoses
MP:0004983	Abnormal osteoclast cell number
ZP:0006539	Abnormal(ly) decreased process quality ossification
MP:0002896	Abnormal bone mineralization
MP:0005006	Abnormal osteoblast physiology

**Table 2:** Summary of OA sub-networks generated by PhenomeExpress and other tools. PhenomeExpress GIGA, JActivemodules and BioNet were used to detect OA related sub-networks from the subchondral bone expression dataset. The number of nodes in each identified sub-network is indicated. For each tool the corresponding statistic value is shown. The most enriched gene ontology biological process relative to the complete network background is shown to indicate the biological function of the sub-networks

PhenomeExpress	Network No.	No. of Nodes	Empirical P value	Top GO Biological Process Annotation
	1	10	1.9E-2	Antigen processing and presentation of exogenous peptide antigen via class II
	2	145	1.0E-4	Immune response
	3	7	4.0E-4	Cell chemotaxis
	4	27	1.0E-4	Defense response to fungi
	5	7	3.0E-2	Lipoxin metabolic process
	6	11	3.1E-3	Extracellular matrix organisation
	7	5	1.4E-3	Regulation of hepatocyte differentiation
	8	5	6.8E-3	Positive regulation of mast cell cytokine production
	9	17	4.3E-2	Transcription DNA-templated
	10	7	3.3E-2	Circadian regulation of gene expression
	11	5	1.4E-2	N/A

GIGA	Network No.	No. of Nodes	Ranked based P-value	Top GO Biological Process Annotation
	1	143	1.2E-205	DNA packing
	2	6	3.6E-9	Neutrophil aggregation
	3	6	2.4E-8	Cell adhesion
	4	5	6.0E-7	Regulation of hepatocyte differentiation
	5	5	1.1E-5	Peptide assembly with MHC protein complex

JactiveModules	Network No.	No. of Nodes	Active Score	Top GO Biological Process Annotation
	1	32	3.1	Antigen processing and presentation of exogenous peptide antigen via MHC class II
	2	31	2.6	Regulation of chromosome segregation
	3	44	3.5	Nucleobase metabolic process
	4	163	5.4	Glyceraldehyde-3-phosphate metabolic process
	5	7	2.4	N/A
	6	8	2.7	Regulation of cytokine mediated signalling pathway
	7	8	2.6	Antigen processing and presentation of exogenous peptide antigen via MHC class II

BioNet	Network No.	No. of Nodes	FDR	Top GO Biological Process Annotation
	1	231	1.0E-5	Negative regulation of cellular macromolecule biosynthetic process

**Table 3:** Phenotypes selected for B-ALL. UberPheno phenotype terms selected for use in the sub-network detection with the PAX5 expression dataset

Phenotype ID	Phenotype Name
HP:0004812	Pre-B-cell acute lymphoblastic leukemia
MP:0012431	Increased lymphoma incidence
HP:0012191	B-cell lymphoma
MP:0008211	Decreased mature B cell number
MP:0008189	Increased transitional stage B cell number

**Table 4:** Summary of B-ALL sub-networks generated by PhenomeExpress and other tools. PhenomeExpress GIGA, JActivemodules and BioNet were used to detect B-ALL related sub-networks from the PAX5 expression dataset. The number of nodes in each identified sub-network is indicated. For each tool the corresponding statistic value is shown. The most enriched gene ontology biological process relative to the complete network background is shown to indicate the biological function of the sub-networks

PhenomeExpress	Network No.	No. of Nodes	Empirical p-value	Top GO Biological Process Annotation
	1	106	1.0E-4	Immune system process
	2	47	1.0E-4	DNA metabolic process
	3	14	1.2E-2	Steroid metabolic process
	4	11	2.6E-2	Protein processing
	5	22	7.4E-3	Protein glycosylation
	6	7	2.7E-2	Complement activation, classical pathway
	7	6	3.4E-2	Polyamine metabolic process
	8	11	1.4E-3	Muscle contraction

GIGA	Network No.	No. of Nodes	Rank based p-value	Top GO Biological Process Annotation
	1	93	4.0E-137	DNA replication
	2	19	2.6E-27	Positive regulation of immune system process
	3	13	1.5E-19	G-protein coupled receptor signaling pathway
	4	14	8.0E-16	Activation of immune response
	5	8	9.5E-10	Marcomolecule glycosylation
	6	10	1.8E-9	Marcomolecule glycosylation
	7	11	3.8E-7	Cation transport
	8	8	3.9E-7	Pyroptosis
	9	11	2.5E-6	Cholesterol metabolic process
	10	8	2.0E-5	Protein localization

JactiveModules	Network No.	No. of Nodes	Active Score	Top GO Biological Process Annotation
	1	151	9.3	Cell Cycle
	2	18	2.2	Sphingolipid metabolic process
	3	9	2.4	Pyroptosis
	4	8	3.8	Cell Cycle
	5	6	3.8	Mitotic cell cycle process
	6	6	3.9	DNA replication
	7	7	3.9	Mitotic nuclear division
	8	6	3.3	N/A
	9	5	3.6	DNA repair
	10	10	1.8	G-protein coupled receptor signalling pathway
	11	102	5.5	Mitotic cell cycle
	12	8	4.0	N/A
	13	15	4.5	DNA repair
	14	12	5.0	Ribonucleoprotein complex biogenesis
	15	42	5.8	DNA metabolic process
	16	5	2.6	DNA replication
	17	8	7.0	Ribosome biogenesis
	18	6	2.1	Mitotic cell cycle phase transition
	19	109	3.5	Cell cycle
	20	9	3.6	DNA repair
	21	5	3.6	DNA unwinding involved in DNA replication
	22	5	3.5	Chromatin assembly
	23	7	3.6	Intracellular transport
	24	12	3.7	Ribosome biogenesis

BioNet	Network No.	No. of Nodes	FDR	Top GO Biological Process Annotation
	1	124	1.0E-25	DNA replication
