# Supplementary material for: PhenomeExpress: A refined network analysis of expression datasets by inclusion of known disease phenotypes
Source: Sci Rep. 2015 Jan 29;5:8117. doi: 10.1038/srep08117 (PMC4822650; doi:10.1038/srep08117)
Supplement: Supplementary Information — Supplementary Figure S1 [file srep08117-s1.pdf]

Supplementary Information for:

PhenomeExpress: A refined network analysis of expression datasets by inclusion of known disease phenotypes

Jamie Soul, Timothy E. Hardingham,Raymond P. Boot-Handford, Jean-Marc Schwartz

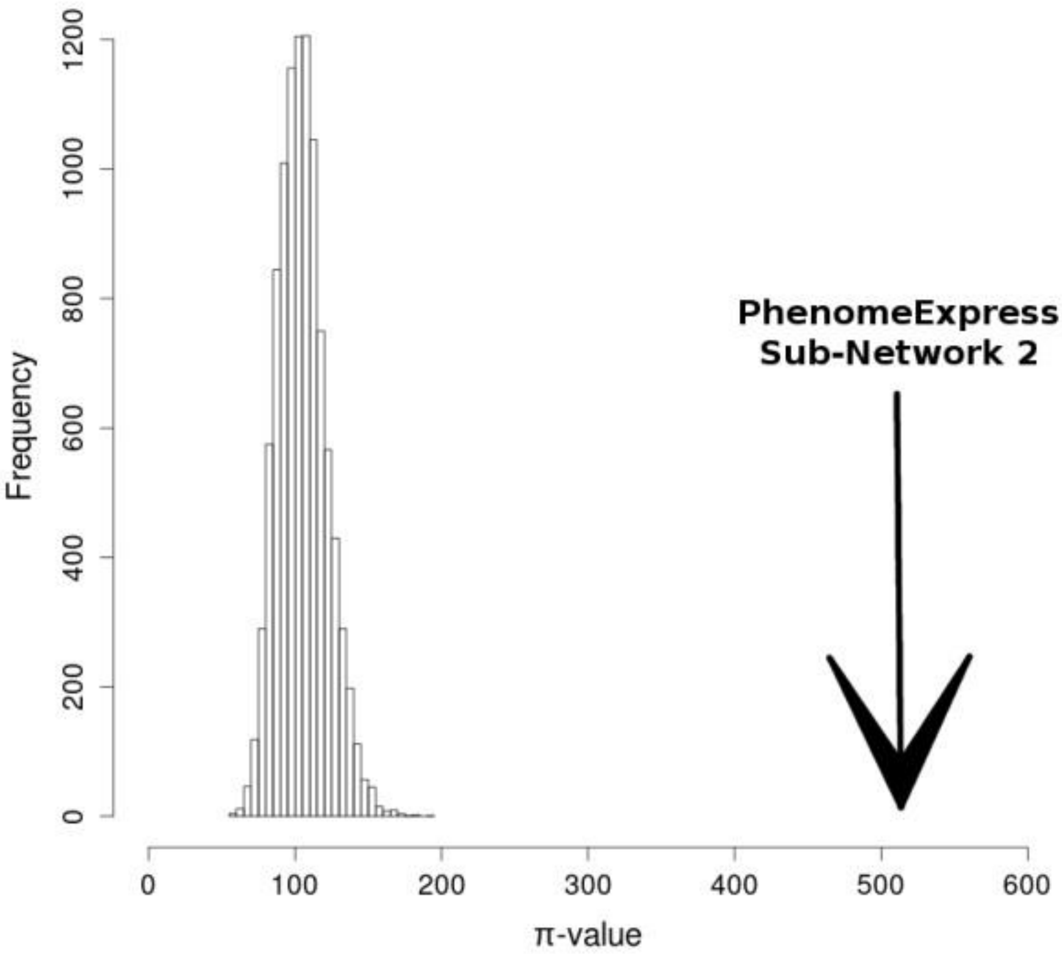

Supplementary Figure S1 – FDR analysis of PhenomeExpress sub-network 2 from the subchondral bone dataset.

Histogram showing the sum of π-values for 10,000 random sub-networks of size equal to the size of sub-network 2 (145 nodes).
